# Castleman disease mimicking accessory spleen on imaging: A case report

**DOI:** 10.1016/j.radcr.2024.09.143

**Published:** 2024-11-11

**Authors:** Niloofar Ayoobi Yazdi, Arman MomeniAmjadi, Rad ghannadzadeh kermanipour, Sajjad Alizadeh, Faeze Salahshour, Mohammadreza Tahamtan

**Affiliations:** a Advanced Diagnostic and Interventional Radiology Research Center (ADIR), Tehran University of Medical Sciences, Tehran, Iran; b School of Medicine, Tehran University of Medical Sciences, Tehran, Iran; c Department of Pathology, School of medicine, Tehran University of Medical Sciences, Tehran, Iran

**Keywords:** Castleman disease, Computed tomography, Magnetic resonance imaging, Accessory spleen

## Abstract

Castleman disease (CD) is a nonclonal lymphoproliferative disorder that causes non-neoplastic lymph node enlargement. With an incidence of approximately 21-25 cases per million, CD presents variably, often mimicking both benign and malignant conditions across various body regions. Clinically, it ranges from asymptomatic lymph node enlargement in Unicentric Castleman's Disease (UCD) to aggressive, multicentric presentations affecting multiple organs. Accurate diagnosis relies on surgical pathology due to the disease's diverse clinical and imaging manifestations. We report a rare case of UCD in a 19-year-old male who presented with mild, nonspecific left upper quadrant pain. Initial examinations, including ultrasonography, computed tomography, and magnetic resonance imaging, showed a hypervascular retroperitoneal mass that was initially suspected to be an accessory spleen or pancreatic tail neuroendocrine tumor.

Surgical resection and histopathological analysis established the diagnosis of hyaline-vascular type UCD. This case highlights the diagnostic challenges of UCD, particularly when presented in uncommon locations like the retroperitoneal peripancreatic region. Imaging often fails to conclusively differentiate CD from other vascular lesions, necessitating a histopathological evaluation. Prior case studies have also reported similar diagnostic challenges and the efficacy of surgical resection for treating UCD. This case report adds to the existing literature by outlining the diagnostic procedure and challenges associated with retroperitoneal UCD. This highlights the need for increased awareness, advanced imaging techniques, and histopathological confirmation to achieve accurate diagnosis and effective treatment. A multidisciplinary approach is critical in managing such complex cases, ultimately leading to favorable patient outcomes.

## Introduction

Castleman disease is a nonclonal lymphoproliferative disorder that causes non-neoplastic lymph node enlargement. The incidence of CD is approximately 21-25 cases per million, with a slight male predominance [1].

Due to its varied presentations and potential to impact any region of the body, CD often mimics both benign and malignant conditions in the neck, chest, abdomen, and pelvis [2]. Histologically, CD can be classified into 3 groups: hyaline-vascular (HV), plasma cell (PC), and mixed-type [3]. Clinically, this disease presents with a spectrum of manifestations, from asymptomatic lymph node enlargement, which is called Unicentric Castleman's Disease (UCD), to a more aggressive presentation, which includes recurrent episodes of widespread lymph node enlargement and multiorgan involvement, called Multicentric Castleman's Disease [4,5]. Clinical findings may range from isolated pain or symptoms related to mass effects, such as abdominal pain, dyspnea, or compression of contiguous structures [6,7].

Imaging findings of this disease can be confused with other conditions, leading to potential misdiagnosis and differences in treatment plans. A definitive diagnosis can only be made through surgical pathology [8,9].

The present case report highlights a very rare case of unicentric Castleman disease, which presents as hypervascular mass in the retroperitonea. This underlines the challenges in the diagnosis of this rare condition and also emphasizes the need for a multidisciplinary approach to the right diagnosis and appropriate management.

## Case Presentation

A 19-year-old male patient presented with nonspecific mild left upper quadrant pain. He had no significant medical history and did not mention any associated symptoms. The patient was a nonsmoker and did not consume alcohol. Physical examination revealed no positive signs. The patient laboratory data are summarized in Table 1.

**Table 1:** The patient's laboratory results.

Parameter	Value	Normal Range
Red Blood Cells (RBC)	5.27	4.7-6.1 million cells/µL
White Blood Cells (WBC)	6.96	4.0-11.0 thousand cells/µL
Hemoglobin (HGB)	15.2	13.8-17.2 g/dL
Hematocrit (HCT)	45	40.7%-50.3%
Neutrophils (Neut)	65.9%	40%-60%
Lymphocytes (Lymph)	24.1%	20%-40%
Monocytes (Mono)	6.1%	2%-8%
Eosinophils (Eos)	1.7%	1%-4%
Aspartate Aminotransferase (AST)	18	10-40 U/L
Alanine Aminotransferase (ALT)	17	7-56 U/L
Alkaline Phosphatase (ALP)	210	44-147 U/L
Gamma-Glutamyl Transferase (gGT)	23	9-48 U/L
Total Bilirubin	0.92	0.1-1.2 mg/dL
Direct Bilirubin	0.35	0.0-0.4 mg/dL
Lactate Dehydrogenase (LDH)	240	140-280 U/L

The initial abdominal ultrasound examination revealed a hypoechoic round mass measuring approximately 45 × 55 mm, anterior to the pancreatic tail and medial to the splenic hilum. In color Doppler mode, the mass shows significant vascularity. Differential diagnosis as accessory spleen, pancreatic tail tumors like neuroendocrine tumor or lymphoma were mentioned.

**Fig. 1:**
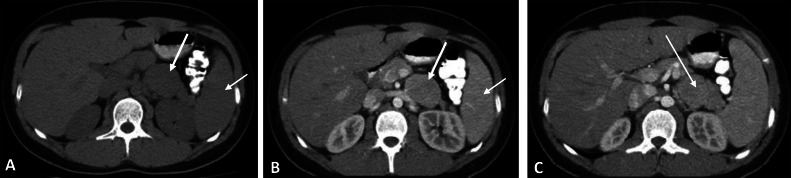
Abdominal CT scan without contrast (A) revealed a homogenous density retroperitoneal mass(arrows) adjacent to the pancreatic tail. Contrast-enhanced imaging (B, C) showed a similar enhancement pattern to the spleen, suggesting an accessory spleen as a potential differential diagnosis.

To assess the origin of the tumor and evaluate the other organs in the abdomen, CT scan with IV contrast is performed. CT scan shows a retroperitoneal mass adjacent to the pancreatic tail shows a similar enhancement pattern to the spleen in the arterial and portal phases. However, to confirm the diagnosis of accessory spleen and exclude neuroendocrine tumor of the pancreatic tail, a Tc99m-denatured RBC Scan and octreotide scintigraphy are recommended (Fig. 1).

**Fig. 2:**
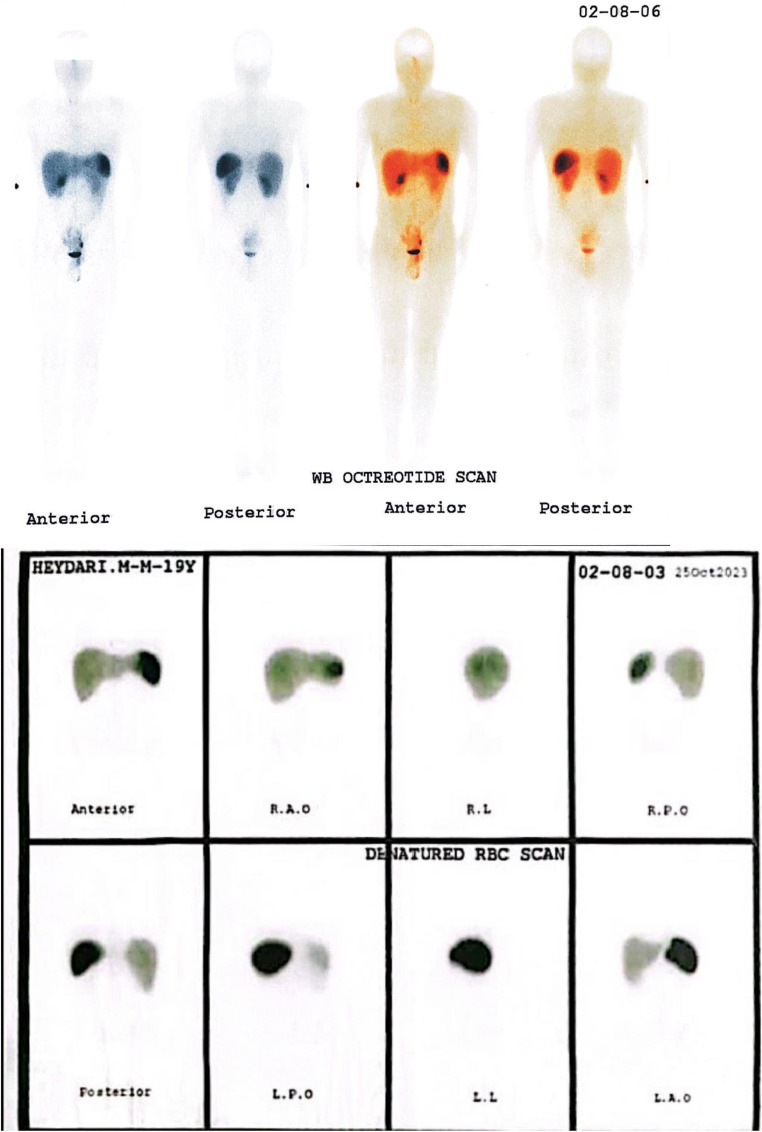
Octreotide scintigraphy (left side) and denaturated RBC scan (right side) revealed no uptake, excluding neuroendocrine tumor of the pancreatic tail and accessory spleen.

Tc99m-denatured RBC Scan and octreotide scintigraphy showed no uptake in the upper quadrant (pancreatic tail to splenic hilum). This finding suggested that the lesion did not originate from the spleen or represent a neuroendocrine tumor (Fig. 2).

**Fig. 3:**
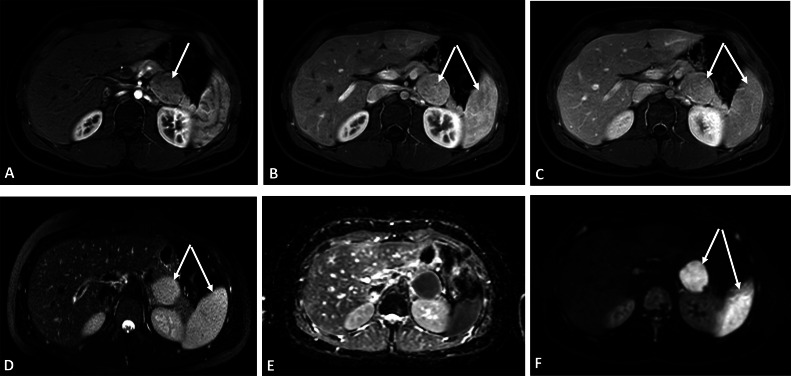
Abdominal MRI with IV contrast demonstrated heterogeneous typical enhancement of the spleen in T1-weighted axial contrast-enhanced early arterial phase images (A), with similar enhancement observed in the retroperitoneal mass(arrow). Late arterial and portal phases also showed enhancement of the lesion comparable to the spleen (B, C). The lesion appeared as a hypersignal mass in T2-weighted images (arrow) (D). In the ADC (Apparent diffusion coefficient) map and DWI (Diffusion-weighted imaging) sequences (E, F), the lesion showed a high diffusion signal similar to the spleen.

Due to a discrepancy between previous modalities, MRI with IV contrast was performed to further evaluation (Fig. 3). In MRI images the lesion exhibits features similar to the spleen.

The multidisciplinary team decided to perform endoultrasonography and biopsy.

The EUS report described a well-demarcated hypervascular solid mass measuring 50 × 45 mm in close contact with the left kidney. The pancreas appeared intact, indicated that the lesion did not originate from the pancreas. An EUS-guided biopsy of the lesion was performed, and histopathological analysis revealed lymphoid tissue.

**Fig. 4:**
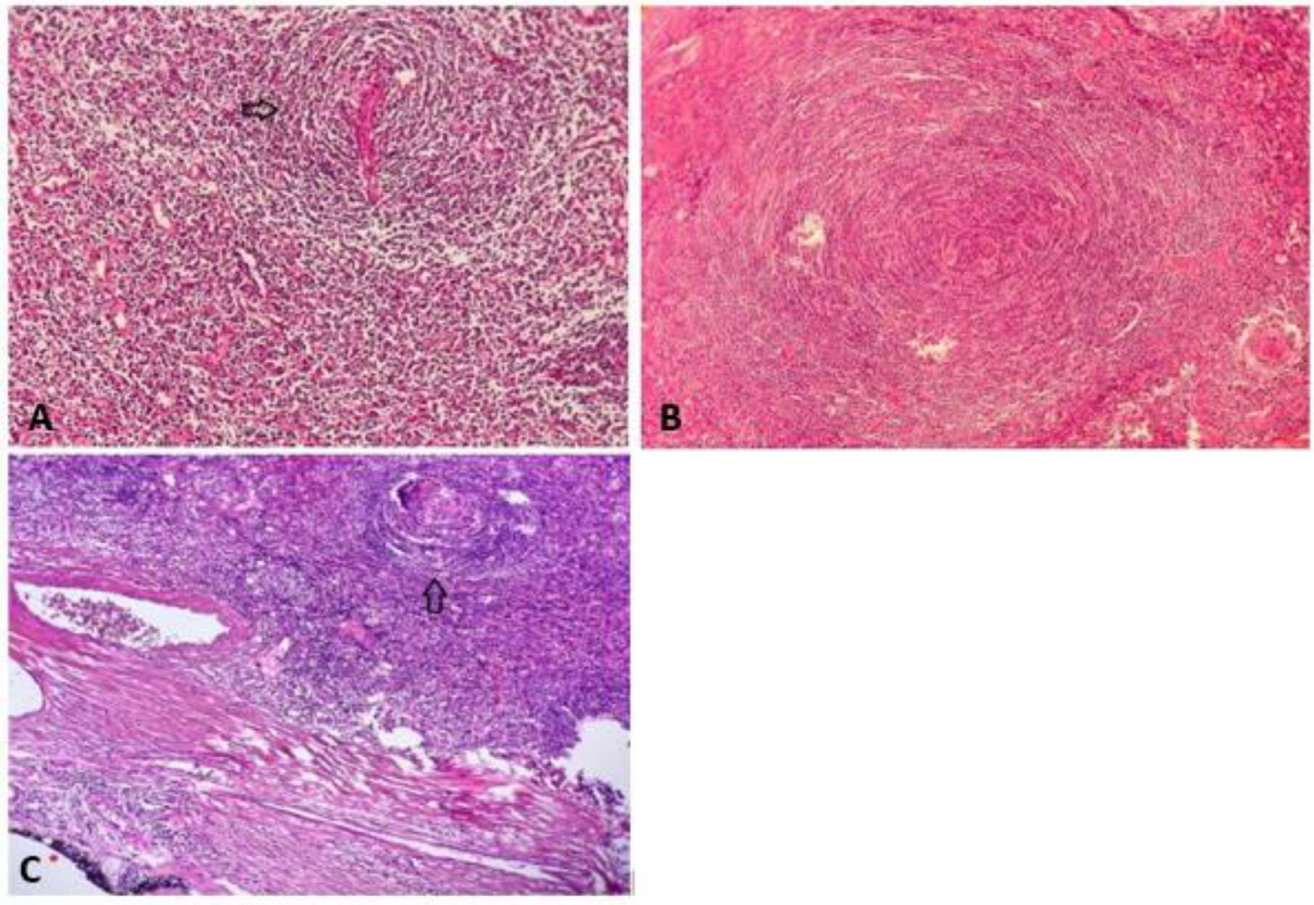
Histologic section of the retroperitoneal mass. (A) Germinal centers traversed by sclerotic penetrating vessels and hyalinization-lollipop follicles (arrow). (B) Mantle zones are thickened with lymphocytes arranged in layers - onion skin appearance. (C) The inked abdominal mass margin is obvious (*) with muscle fibers and vessels beneath it. Follicle with features of Castleman disease (arrow).

Based on the diagnostic workup, the patient underwent surgical resection of the lesion. The mass was completely excised, and the postoperative pathological examination revealed features consistent with Castleman disease (hyaline-vascular type) (Fig. 4).

## Discussion

Castleman disease (CD) presented with various clinical and radiological manifestations, poses a significant diagnostic challenge. In the mentioned case, we describe an unusual manifestation of UCD as a retroperitoneal enhancing mass, mimicking an accessory spleen on imaging, emphasizing the difficulties of diagnosing this disease. UCD can affect all lymph nodes, particularly those in the mediastinum, neck, retroperitoneum, and axillary regions. However, its presence in the retroperitoneal peripancreatic area is less common [10].

CD often presents as a well-defined mass with inhomogeneous enhancement on contrast-enhanced imaging, which can be mistaken with other vascular lesions, such as neuroendocrine tumors, paraganglioma, lymphoma [11], and, in our case, an accessory spleen.

In this case, the patient's nonspecific LUQ pain and unspecific clinical data complicated the diagnosis. Imaging played a major role in the diagnostic process. Imaging modalities suggested a retroperitoneal peripancreatic mass with similar features as the spleen, which suggests accessory spleen (splenole) as the most probable diagnosis. However, absence of the Tc99m-denatured RBC Scan uptake did not confirm the diagnosis of accessory spleen. Moreover, because of the significant enhancement of the lesion located near the tail of the pancreas, neuroendocrine tumor was also considered initially, but further imaging and octreotide scan showed the lesion did not originate from the pancreas tail. Lymphoma was not compatible with the imaging features of the patient, because of the absence of hepatosplenomegaly, para-aortic lymphadenopathy, and peritoneal, mesenteric abnormalities. Moreover, lymphoma typically shows less enhancement than what was observed in this case [12].

Most UCD lesions show inhomogeneous enhancement on enhanced CT images. Additionally, some tumors have a rich vascular supply and are accompanied by microcalcifications . On MRI, most retroperitoneal UCD lesions appear isointense or hypointense on T1-weighted images (T1WI) and slightly hyperintense or hyperintense on T2-weighted images (T2WI) [8]. Although the enhancement characteristics are similar in both CT and MRI, MRI is better than CT at identifying the relationship between the mass and adjacent vessels or tissues . Furthermore, ultrasound is advantageous in assessing the location of the mass and its nearby feeding vessels [13]. These findings are consistent with the radiological features observed in our patient on both CT and MRI. (Fig. 1, Fig. 3)

The imaging manifestations of UCD are challenging to differentiate from other conditions, and the preoperative imaging findings often do not align with the postoperative histopathological diagnosis [14], therefore the diagnosis of UCD still relies on histopathological examination [10].

The definitive diagnosis of CD, specifically the hyaline-vascular type in this case, was established through histopathological study following the surgical resection.

Prior case reports and reviews have identified the multiple manifestations of CD. For instance, Cheng et al. described similar diagnostic challenges in distinguishing retroperitoneal UCD from neuroendocrine tumor [8].Stevens et al. described a CD resembling a spinal nerve sheath tumor in the central nervous system diagnosed postoperatively [11]. Bonekamp et al. mentioned that CD can mimic lymphoma, metastatic adenopathy, and infectious and/or inflammatory diseases that result in adenopathy [2] and in this case, we presented a CD mimicing accessory spleen.

This case highlights the necessity of considering CD in the differential diagnosis for a retroperitoneal enhancing mass. UCD is typically well-localized and can be treated successfully with complete surgical resection of the tumor. This remains the most effective treatment for retroperitoneal UCD, with low recurrence and high cure rates [15].

A multidisciplinary approach is required for precise diagnosis and successful treatment of CD. To achieve this goal, cooperation between radiologists, pathologists, surgeons, and oncologists is required to meet comprehensive investigation and appropriate treatment planning. This approach is particularly noticeable regarding CD's ability to mimic other conditions and the importance of histopathological studies for the diagnosis [[3], [4], [5]].

This case report adds further information to the literature regarding the diagnostic challenges of CD. Advanced imaging methods and the importance of histopathological evaluation play pivotal roles in the diagnosis. Early detection and suitable treatment of UCD can lead to promising results, which emphasizes the consideration of CD in the differential diagnosis.

Describing the diagnostic process, imaging features, histopathological studies, and clinical management of this specific case will help other physicians better manage similar cases with similar presentations to yield the best outcomes.

## Conclusion

This case report contributes to the existing literature by detailing the diagnostic process and challenges linked to retroperitoneal UCD. It emphasizes the importance of increased awareness, advanced imaging methods, and histopathological confirmation for accurate diagnosis and effective treatment. A multidisciplinary approach is essential to exclude other conditions with similar presentations, as Castleman disease can mimic other abnormalities.

## Patient consent

Written, informed consent was obtained from the patient for publication of this case.

## Ethics approval

This study was performed in line with the principles of the Declaration of Helsinki.

The authors affirm the patient provided informed consent for publication of the images in Fig. 1, Fig. 2, Fig. 3.
